# Synthesis and pharmacological effects of novel benzenesulfonamides carrying benzamide moiety as carbonic anhydrase and acetylcholinesterase inhibitors

**DOI:** 10.3906/kim-2007-37

**Published:** 2020-12-16

**Authors:** Mehtap TUĞRAK, Halise İnci GÜL, Barış ANIL, İlhami GÜLÇİN

**Affiliations:** 1 Department of Pharmaceutical Chemistry, Faculty of Pharmacy, Atatürk University, Erzurum Turkey; 2 Department of Chemistry, Faculty of Science, Atatürk University, Erzurum Turkey

**Keywords:** Sulfonamide, benzamide, carbonic anhydrase, acetylcholinesterase, enzyme inhibition

## Abstract

*N* -(1-(4-Methoxyphenyl)-3-oxo-3-((4-( *N* -(substituted)sulfamoyl)phenyl)amino)prop-1-en-1-yl)benzamides **3a – g** were designed since sulfonamide and benzamide pharmacophores draw great attention in novel drug design due to their wide range of bioactivities including acetylcholinesterase (AChE) and human carbonic anhydrase I and II (hCA I and hCA II) inhibitory potencies. Structure elucidation of the compounds was carried out by 1H NMR, 13C NMR, and HRMS spectra. In vitro enzyme assays showed that the compounds had significant inhibitory potential against hCA I, hCA II, and AChE enzymes at nanomolar levels. Ki values were in the range of 4.07 ± 0.38 – 29.70 ± 3.18 nM for hCA I and 10.68 ± 0.98 – 37.16 ± 7.55 nM for hCA II while Ki values for AChE were in the range of 8.91 ± 1.65 – 34.02 ± 5.90 nM. The most potent inhibitors **3g** (Ki = 4.07 ± 0.38 nM, hCA I), **3c** (Ki = 10.68 ± 0.98 nM, hCA II **)** , and **3f** (Ki = 8.91 ± 1.65 nM, AChE) can be considered as lead compounds of this study with their promising bioactivity results. Secondary sulfonamides showed promising enzyme inhibitory effects on AChE while primary sulfonamide derivative was generally effective on hCA I and hCA II isoenzymes.

## 1. Introduction

Owing to significant bioactivities of benzamide pharmacophores, this group of compounds has been used for designing novel and effective bioactive compounds. Benzamide and its derivatives have been reported with antimicrobial, analgesic anticancer, carbonic anhydrase inhibitory, cholinesterase inhibitory activities and so on [1–4].

One of the diseases that affects elderly people is Alzheimer’s disease (AD) which is a neurodegenerative disease that causes progressive dementia [5,6]. Moreover, it is a global economic and social burden. According to the World Alzheimer Report, it is estimated that there will be around 131.5 million cases by 2050 [7]. Some of the pathologies of AD are the loss of cholinergic neurons and a decreasing amount of neurotransmitter acetylcholine (ACh) in synapses. Cholinesterases [acetylcholinesterase (AChE) and butyrylcholinesterase (BuChE)] have a crucial role in the process of AD that regulates synaptic levels of Ach [8]. Cholinergic hypothesis was the first approach to explain the symptoms of AD. While AChE levels in the brain reduce, the activity of BuChE either does not change, or in some cases increases. Therefore, inhibiting AChE activity could be a beneficial pathway as a disease-modifying aspect [9,10]. AChE inhibitors are the most used medication in the treatment of AD which act as irreversible or reversible inhibitors. While donepezil and galantamine are reversible inhibitors, rivastigmine is a pseudo-irreversible inhibitor. Current treatments used in the clinic unfortunately cannot prevent the progression of AD but they can temporarily relieve symptoms, or decelerate the progress. Even though some valuable progress has been made in the treatment of AD, we still urgently need to find out more selective and effective antiAD drugs with less side effects than the ones available on the market [11].

As one of the most studied enzyme families that target many diseases, carbonic anhydrases (CAs, EC 4.2.1.1) are zinc-binded enzymes that catalyze the reversible hydration of CO2. CA isoenzymes play an important role in many physiological and pathological processes such as tumorigenicity, respiration, regulation of pH, electrolytes secretion, biosynthesis of membrane lipids, and nucleotides. Among the most studied isotypes of CAs, cytosolic CA I and CA II isoenzymes act as targets for glaucoma and epilepsy while CA IX and CA XII are promising well-known targets for current anticancer research [12–16].

Since some of CAs isoenzymes have similar protein structures and distribution in the body, selective inhibition of them is the major concern of the researchers [17]. As CA I and CA II isoenzymes are widespread enzymes, their nonselective inhibition may cause several side effects [18, 19].

Different kinds of zinc binding compounds have been recognized as compounds targeting CAs [20]. Sulfonamides are powerful CAs inhibitors which show their activity by binding in an anion form with the zinc ion at the active site of the enzyme [21]. Primary and secondary sulfonamide and their analogs show significant CAs inhibitory potency. The most popular CAs inhibitors used in clinics for decades are acetazolamide, methazolamide, ethoxzolamide, dorzolamide, and brinzolamide [21–23]. In addition, sulfonamide derivatives are also known to have a wide-range of bioactivities, such as diuretic, antiglaucoma, anticancer, and antioxidant. Their significant pharmacological activities have also led to the design of new attractive candidates to be evaluated in clinical studies [22,24].

**Figure 1:**
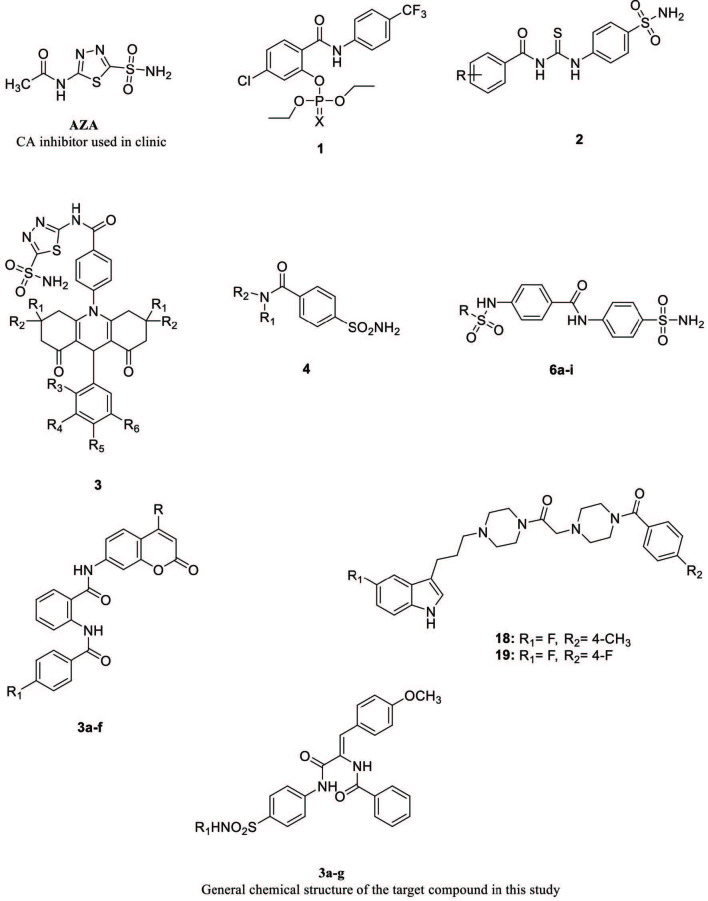
Benzamide derivatives reported as AChE and CAs inhibitors.

Current studies indicate that benzamide bearing benzene sulfonamides showed inhibitory potency against CA I, CA II, and AChE enzymes (Figure 1). 2-Hydroxy- *N* -phenylbenzamides 1 (Figure 1) were reported as novel inhibitors of cholinesterases [11]. The benzamides moderately inhibited AChE with IC50 values in the range of 33.1 to 85.8 µM while their IC50 values for BuChE were in the range of 53.5–228.4 µM. Most of the compounds studied in literature inhibited AChE more efficiently than BuChE [11]. In a study, sulfonamide and its derivatives bearing benzamide moiety 2 (Figure 1) were declared as potent inhibitors of hCA II with IC50 values 0.09–0.58 µM [21]. 4-Amino-N-(5-sulfamoyl-1,3,4-thiadiazol-2-yl)benzamide derivatives 3 were investigated as inhibitors of CAI, CAII, CAVII, and CA IV in another study. Ki values of the compounds mentioned on hCA I and II were in the range of 6.7–335.2 nM (hCA I) and 0.5–55.4 nM (hCA II) while hCA IV and VII were inhibited with Ki values in the range of 29.7–708.8 nM (hCA IV), and of 1.3–90.7 nM (hCA VII) [25]. In a different study, benzamides bearing 4-sulfamoyl moieties 4 inhibited the hCA II, VII, and IX in the nanomolar ranges [26]. In another study, novel sulfonamide derivatives 6a–i (Figure 1), as novel carbonic anhydrase inhibitors which are candidates for glaucoma treatment were synthesized. In vitro results showed that the novel compounds had a significant inhibitory profile against hCA I and hCA II enzymes at the nanomolar levels. Ki values were in the range of 0.020 ± 0.0003 – 0.065 ± 0.0005 nM for hCA I and 0.011 ± 0.0001 – 0.045 ± 0.0004 nM for hCA II [27].

Bozdag M et al. reported CAs inhibitory studies of 2-benzamido-N-(2-oxo-4-(methyl/trifluoromethyl)-2 *H* -chromen-7-yl) benzamide 3a–f (Figure 1), as selective inhibitors of the tumor associated with hCA IX and XII isoforms. Among the compounds reported, the trifluoromethyl derivative 3d was the most potent against these CA isoforms with Ki valuess of 10.9 and 6.7 nM [28]. In a different study, new series of indolylpropyl-piperazinyl oxoethyl-benzamido piperazines were synthesized and evaluated as multitarget-directed drugs for acetylcholinesterase (AChE). In vitro results showed that at least four compounds displayed promising activity against AChE. Compounds 18 and 19 (Figure 1), (IC50 = 3.4 and 3.6 mM respectively) exhibited AChE inhibition profile in the same order of magnitude as donepezil (DPZ, IC50 = 2.17 mM) [29].

In light of the previous studies summarized above, in this study, *N* -(1-(4-methoxyphenyl)-3-oxo-3-((4-( *N* -(substituted)sulfamoyl)phenyl)amino)prop-1-en-1-yl)benzamides 3a–g having benzamide and primary or secondary sulfonamides pharmacophors were designed and their effects on hCA I, hCA II, and AChE enzymes were investigated in order to find out novel and effective enzyme inhibitors.

## 2. Materials and methods

### 2.1. Chemistry

1H NMR (400MHz) and 13C NMR (100 MHz) (Varian Mercury Plus spectrometer, Varian Medical Systems, Inc., Palo Alto, CA, USA) and HRMS (Shimadzu Corp., Kyoto, Japan) spectral techniques were used for the confirmation of chemical structures of the compounds. Chemical shifts (δ) are reported in ppm, and coupling constants ( *J* ) are expressed in hertz (Hz). Electrothermal 9100/IA9100 instrument (Bibby Scientific Limited, Staffordshire, UK) was used to find melting points. Thin layer chromatography (TLC) using silica gel 60 HF254 (Merck KGaA, Darmstadt, Germany) was used to check the reaction process. Chloroform: methanol (4.8:0.2) solvent mixture was used as TLC solvent system.

### 2.2. Synthesis of 2-benzamidoacetic acid (1) and 4-(4-methoxybenzylidene)-2-phenyloxazol-5(4H)-one (2) (Figure 2)

**Figure 2:**
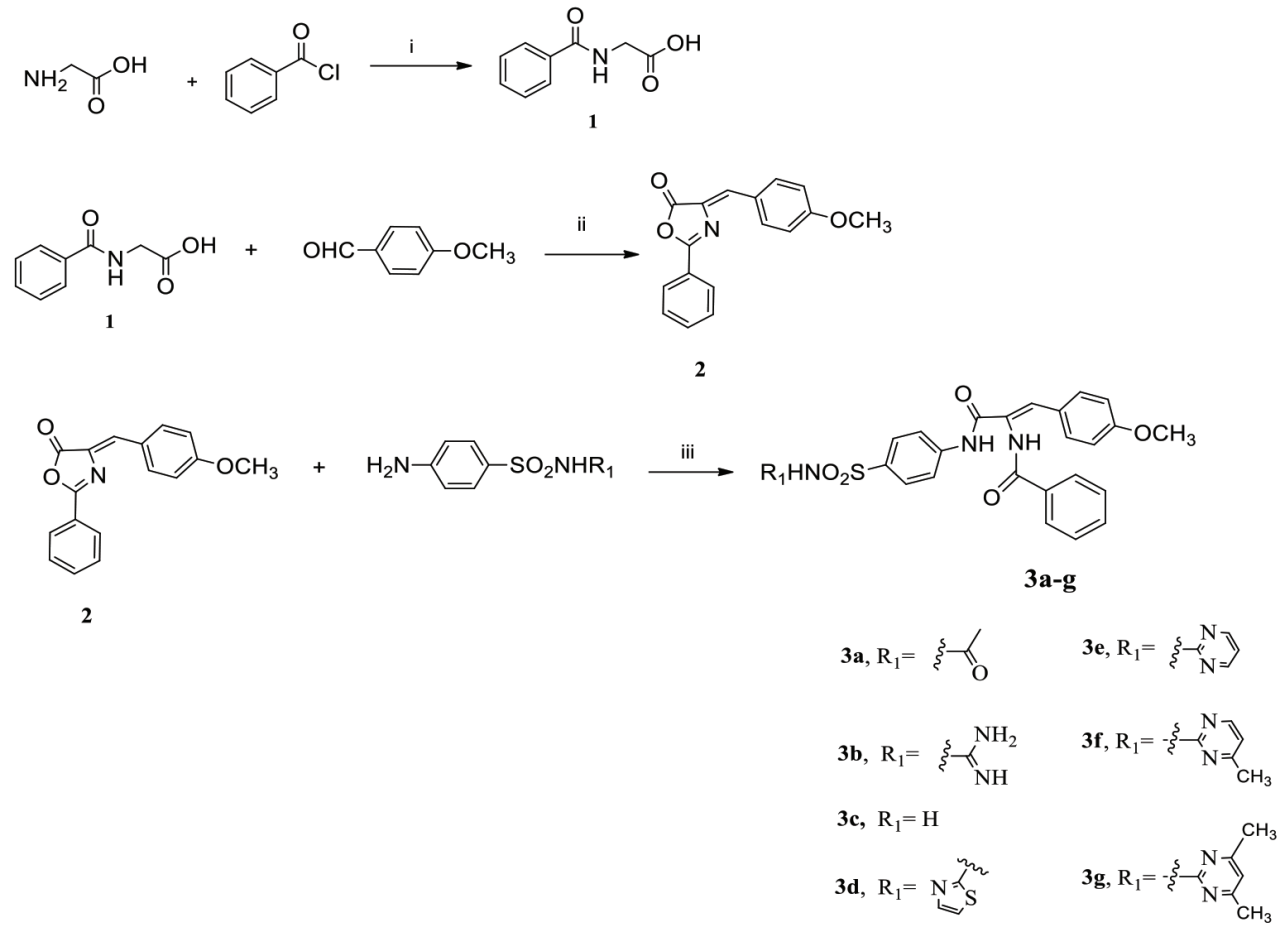
Synthesis of target compounds 3a–g. Reagents and reaction conditions: i: 10% NaOH, HCl, ii: acetic anhydride, sodium acetate, 100 ° C, iii: glacial acetic acid, sodium acetate, 100 °C

The compounds 1 and 2 were reported previously in literature [23,30]. Chemical structures of the compounds were confirmed with 1H NMR, 13C NMR, and HRMS spectral techniques in this study. Data were not added here for registered compounds.

### 2.3. General synthesis method of 3a–g

A mixture of compound 2 (10 mmol), a suitable sulfonamide derivative (12 mmol) [sulfacetamide (3a), sulfaguanidine (3b), sulfanilamide (3c), sulfathiazole (3d) sulfadiazine (3e), sulfamerazine (3f), sulfamethazine (3g)] and sodium acetate (15 mmol) in glacial acetic acid (10 mL) was refluxed at 100 ºC for 18–22 h (3a (20 h), 3b (22 h), 3c (18 h), 3d (21 h), 3e (19 h), 3f (20 h), 3g (19 h)). The reaction process was monitored by TLC. The product was filtered off, washed with water, and dried. It was recrystallized from DMF / C2H5OH / H2O [23]. Compounds 3a–g were synthesized successfully for the first time (except compounds 3c and 3d) according to Figure 2 [31]. Experimental data on compounds 3c and 3d have not been presented in the literature. The spectrum data (1H NMR, 13C NMR, and HRMS) of all compounds (3a–g) are given below; at the same time all of the chemistry data were also given as supporting information.

### 2.4. Spectral data of the compounds 3a-g

#### 2.4.1. N-(3-((4-(N-acetylsulfamoyl)phenyl)amino)-1-(4-methoxyphenyl)-3-oxoprop-1-en-1-yl)benzamide (3a)

Cream color solid, mp = 209–210 °C. Yield 65%. 1H NMR spectrum, (DMSO - *d* *6* , 400 MHz), *δ* , ppm ( *J* , Hz): 12.01 (1H, s, NH); 10.54 (1H, s, NH); 10.12 (1H, s, NH); 8.05 (2H, d, *J* = 7.2 Hz, ArH); 7.94 (2H, d, *J* = 8.9 Hz, ArH); 7.86 (2H, d, *J* = 8.9 Hz, ArH); 7.65–7.59 (2H, m, ArH); 7.56–7.52 (3H, m, ArH); 7.17 (1H, s, =CH-); 6.99 (2H, d, *J* = 7.2 Hz, ArH); 3.77 (3H, s, -OCH3); 1.90 (3H, s, -CH3). 13C NMR spectrum, (DMSO - *d* *6* , 100 MHz), *δ* , ppm: 168.9; 165.9; 165.1; 159.7; 158.1; 137.2; 133.3; 131.8; 131.3; 129.1; 128.6; 128.44; 128.37; 127.9; 126.4; 119.3; 114.1; 55.2; 23.3. HRMS, found m/z: 494.1388 [M + H] +. C25H23N3O6S. Calculated m/z: 494.1380.

#### 2.4.2. N-(3-((4-(N-(diaminomethylene)sulfamoyl)phenyl)amino)-1-(4-methoxyphenyl)-3-oxoprop-1-en-1-yl)benzamide (3b)

Dark cream color solid, mp = 278–280 °C. Yield 63%. 1H NMR spectrum, (DMSO - *d* *6* , 400 MHz), *δ* , ppm ( *J* , Hz): 10.39 (1H, s, NH); 10.09 (1H, s, NH); 8.05 (2H, d, *J* = 7.9 Hz, ArH); 7.84 (2H, dd, *J* = 8.5 Hz, *J* = 1.3 Hz, ArH); 7.71 (2H, dd, *J* = 8.7 Hz, *J* = 1.6 Hz, ArH); 7.64–7.59 (2H, m, ArH); 7.56–7.52 (3H, m, ArH); 7.17 (1H, s, =CH); 6.98 (2H, d, *J* = 7.8 Hz, ArH); 6.69 (bs, 4H, NH2); 3.77 (s, 3H, -OCH3). 13C NMR spectrum, (DMSO - *d* *6* , 100 MHz), *δ* , ppm: 165.91; 164.85; 159.7; 158.0; 141.8; 138.8; 133.4; 131.8; 131.3; 128.9; 128.5; 128.4; 127.9; 126.5; 126.3; 119.3; 114.0; 55.2. HRMS, found m/z: 494.1505 [M + H] +. C24H23N5O5S. Calculated m/z: 494.1493.

#### 2.4.3. N-(1-(4-methoxyphenyl)-3-oxo-3-((4-sulfamoylphenyl)amino)prop-1-en-1-yl)benzamide (3c)

Light cream color solid, mp = 254–256 °C. Yield 58%. 1H NMR spectrum, (DMSO - *d* *6* , 400 MHz), *δ* , ppm ( *J* , Hz): 10.45 (1H, s, NH); 10.11 (1H, s, NH); 8.05 (2H, d, *J* = 7.6 Hz, ArH); 7.90 (2H, dd, *J* = 8.6 Hz, J= 1.7 Hz, ArH); 7.78 (2H, dd, *J* = 1.9 Hz, *J* = 8.8 Hz, ArH); 7.64–7.60 (3H, m, ArH); 7.56–7.53 (2H, m, ArH); 7.28 (2H, s, NH2); 7.18 (1H, s, =CH-); 6.98 (2H, d, *J* = 7.0 Hz, ArH); 3.77 (3H, s, -OCH3). 13C NMR spectrum, (DMSO - *d* *6* , 100 MHz), *δ* , ppm: 166.4; 165.4; 160.2; 142.8; 138.8; 133.9; 132.3; 131.8; 129.6; 128.96; 128.88; 128.4; 126.96; 126.89; 119.9; 114.6; 55.7. HRMS, found m/z: 452.1293 [M + H] +. C23H21N3O5S. Calculated m/z: 452.1275.

#### 2.4.4. N-(1-(4-methoxyphenyl)-3-oxo-3-((4-(N-(thiazol-2-yl)sulfamoyl)phenyl)amino)prop-1-en-1-yl)benzamide (3d)

Cream color solid, mp = 200 – 201 °C. Yield 60%. 1H NMR spectrum, (DMSO - *d* *6* , 400 MHz), *δ* , ppm ( *J* , Hz): 12.71 (1H, s, NH); 10.44 (1H, s, NH); 10.10 (1H, s, NH); 8.04 (2H, d, *J* = 7.7 Hz, ArH); 7.88 (2H, d, *J* = 8.7 Hz, ArH); 7.76 (2H, d, *J* = 8.7 Hz, ArH); 7.63–7.59 (3H, m, ArH); 7.55–7.52 (2H, m, ArH); 7.25 (1H, d, *J* = 7.5 Hz, ArH); 7.15 (1H, s, =CH-); 6.97 (2H, d, *J* = 8.8 Hz, ArH); 6.82 (1H, d, *J* = 7.2 Hz, ArH); 3.76 (3H, s, -OCH3). 13C NMR spectrum, (DMSO - *d* *6* , 100 MHz), *δ* , ppm: 168.7; 165.9; 164.9; 159.7; 142.6; 133.4; 131.8; 131.3; 128.9; 128.5; 128.4; 127.9; 126.7; 126.4; 126.3; 119.4; 114.0; 108.0; 55.2. HRMS, found m/z: 535.1123 [M + H] +. C26H22N4O5S2. Calculated m/z: 535.1104.

#### 2.4.5. N-(1-(4-methoxyphenyl)-3-oxo-3-((4-(N-(pyrimidin-2-yl)sulfamoyl)phenyl)amino)prop-1-en-1-yl)benzamide (3e)

Light yellow color solid, mp = 242–244 °C. Yield 63%. 1H NMR spectrum, (DMSO - *d* *6* , 400 MHz), *δ* , ppm ( *J* , Hz): 11.72 (1H, s, NH); 10.50 (1H, s, NH); 10.12 (1H, s, NH); 8.50 (2H, d, *J* = 7.7 Hz, ArH); 8.04 (2H, d, *J* = 7.7 Hz, ArH); 7.95–7.89 (4H, m, ArH); 7.63–7.59 (3H, m, ArH); 7.55–7.51 (2H, m, ArH); 7.14 (1H, s, =CH-); 7.05–7.02 (1H, m, ArH); 6.99–6.96 (2H, m, ArH); 3.76 (3H, s, -OCH3). 13C NMR spectrum, (DMSO - *d* *6* , 100 MHz), *δ* , ppm: 165.9; 165.1; 159.7; 158.3; 156.9; 143.4; 133.3; 131.8; 131.3; 129.4; 128.9; 128.6; 128.5; 128.4; 127.8; 126.4; 119.1; 114.1; 55.2. HRMS, found m/z: 530.1503 [M + H] +. C27H23N5O5S. Calculated m/z: 530.1493.

#### 2.4.6. N-(1-(4-methoxyphenyl)-3-((4-(N-(4-methylpyrimidin-2-yl)sulfamoyl)phenyl)amino)-3-oxoprop-1-en-1-yl)benzamide (3f)

Yellow color solid, mp = 180 – 181 °C. Yield 62%. 1H NMR spectrum, (DMSO - *d* *6* , 400 MHz), *δ* , ppm ( *J* , Hz): 10.47 (1H, s, NH); 10.07 (1H, s, NH); 10.09 (1H, s, NH); 8.05–8.01 (2H, m, ArH); 7.96–7.89 (3H, m, ArH); 7.69–7.59 (5H, m, ArH); 7.55–7.52 (2H, m, ArH); 7.14 (1H, s, =CH-); 7.10–6.93 (2H, m, ArH); 6.88 (1H, d, *J* = 5.1 Hz, ArH); 3.74 (3H, s, -OCH3); 2.29 (3H, s, -CH3). 13C NMR spectrum, (DMSO - *d* *6* , 100 MHz), *δ* , ppm: 166.7; 165.8; 160.4; 157.3; 143.9; 134.1; 132.5; 132.0; 129.8; 129.5; 129.3; 129.2; 129.1; 128.6; 127.1; 119.83; 119.76; 115.2; 114.8; 55.9; 23.9. HRMS, found m/z: 544.1657 [M + H] +. C28H25N5O5S. Calculated m/z: 544.1649.

#### 2.4.7. N-(3-((4-(N-(4,6-dimethylpyrimidin-2-yl)sulfamoyl)phenyl)amino)-1-(4-methoxyphenyl)-3-oxoprop-1-en-1-yl)benzamide (3g)

Dark cream color solid, mp = 229 – 231 °C. Yield 60%. 1H NMR spectrum, (DMSO - *d* *6* , 400 MHz), *δ* , ppm ( *J* , Hz): 11.72 (1H, s, NH); 10.50 (1H, s, NH); 10.12 (1H, s, NH); 8.04 (2H, d, *J* = 7.6 Hz, ArH); 7.95 (2H, d, *J* = 8.8 Hz, ArH); 7.88 (2H, d, *J* = 8.7 Hz, ArH); 7.63–7.60 (3H, m, ArH); 7.58–7.50 (2H, m, ArH); 7.15 (1H, s, =CH-); 6.98–6.96 (2H, m, ArH); 6.76 (1H, s, ArH); 3.76 (3H, s, -OCH3); 2.26 (6H, s, -CH3). 13C NMR spectrum, (DMSO - *d* *6* , 100 MHz), *δ* , ppm: 166.7; 165.7; 160.4; 156.9; 143.8; 134.1; 132.5; 132.0; 129.8; 129.7; 129.2; 129.1; 128.6; 128.7; 127.2; 119.6; 115.2; 114.8; 114.4; 55.9; 23.6. HRMS, found m/z: 558.1804 [M + H] +. C29H27N5O5S. Calculated m/z: 558.1806.

### 2.5. Biological activity assay

CA isoenzymes (CA I and CA II) were purified using a Sepharose-4B-L tyrosine-sulphanilamide affinity chromatography [32]. Activities of CA isoenzymes were determined according to method by Verpoorte et al. [33]. The absorbance differences were recorded at 348 nm. The quantity of protein was determined at 595 nm according to the Bradford method [34]. The experimental procedure was given in detail in our previous papers [12, 35–37]. SSpectrophotometry-based Ellman’s method was used to test the compounds’ AChE inhibitory effects [38,39]. Acetylthiocholine iodide (AChI) was used as a substrate of the reaction. 5,5-dithiobis(2-nitro-benzoic acid) (DTNB) was used for the determination of the AChE activities. An activity (%)-[Compound] graph was drawn to calculate the inhibition effects of the compounds on CAs and AChE. The IC50 values were obtained from activity (%) versus compounds plots. Three different concentrations were used to calculate Ki values. The Lineweaver–Burk plots [40] were drawn, and calculations were realized as described [41].

## 3. Results and discussion

### 3.1. Chemistry

In this study, compounds having the chemical structure of [ *N* -(1-(4-methoxyphenyl)-3-oxo-3-((4-( *N* -(substituted)sulfamoyl)phenyl)amino)prop-1-en-1-yl)benzamide] (3a–g, Figure 2), were designed and successfully synthesized for the first time (except compounds 3c and 3d) by conventional method using 4-(4-methoxybenzylidene)-2-phenyloxazol-5(4H)-one (2) as an intermediate compound in acetic acid-sodium acetate reaction medium [31]. The chemical structures of the final compounds 3a–g and intermediates (compounds 1 and 2) were confirmed using spectral techniques. Primary sulfonamide sulfanilamide (3c) derivative and secondary sulfonamide derivatives as sulfacetamide (3a), sulfaguanidine (3b), sulfathiazole (3d), sulfadiazine (3e), sulfamerazine (3f), and sulfamethazine (3g) were synthesized. The compounds 3a–g were first reported in this study (except compounds 3c and 3d) [31].

NH protons belong to benzamide moieties indicating open-ring forms of the compounds (3a–g, Figure 2) were in the range of 10.54–10.09 ppm and 10.12–10.09 ppm as singlet. -NH2 protons belonging to primary sulfonamide moiety were seen at 7.28 ppm while other -NH protons of the secondary sulfonamides were seen in the range of 12.71–10.39 ppm as singlet. In addition, β-proton of the 3-oxopropen-1-yl chain was observed in the range of 7.18–7.14 ppm for 3a–g. Methoxy protons on phenyl ring was also seen in the range of 3.77–3.76 ppm as a singlet. In addition to 1H NMR, 13C NMR, and HRMS, results also confirmed the proposed chemical structure of the final compounds 3a–g.

Georgey et al. reported several compounds having similar structures with our target compounds in this study. They isolated 1,2,4-trisubstituted imidazolinones by heating 2-substitutedphenyl-4-(substituted benzylidene)oxazole-5(4 *H* )-ones, the appropriate sulfonamide derivative and freshly prepared fused sodium acetate in a boiling water bath while we obtained open chain benzamide derivatives under reflux conditions in glacial acetic acid [23]. According to our TLC, there were more than two main big spots in addition to slight spots. We tried several solvent systems to purify the crude, resulting in DMF-ethanol-water mixture being found as the most suitable crystallization solvent mixture to obtain pure compounds.

### 3.2. Carbonic anhydrase inhibitory effects

The compounds 3a–g were reported for the first time with their enzyme inhibitory potencies on hCA I, hCA II, and AChE enzymes (Table). Our results indicated that 3a–g had effective inhibition profiles against slow cytosolic isoform hCA I, and cytosolic dominant rapid isoenzyme hCA II with inhibition constants in the low nanomolar range 4.07 ± 0.38 – 29.70 ± 3.18 nM, and 10.68 ± 0.98 – 37.16 ± 7.55 nM, respectively by considering Ki values. On the other hand, acetazolamide (AZA) which is used as a clinical drug had Ki values of 30.74 ± 3.52 nM on hCA I, and 22.27 ± 1.56 nM on hCA II. Ki values of the cycylic compound 2 were 50.34 ± 1.59 nM (hCA I) and 62.54 ± 11.51 nM (hCA II).

**Table:** The inhibitory effects of the compounds 3a–g onCAs isoenzymes.

Compounds	IC50						Ki (nM)		
	hCA I(nM)	r2	hCA II(nM)	r2	AChE(nM)	r2	hCA I	hCA II	AChE
2	40.76	0.9828	46.20	0.9773	38.50	0.9921	50.34 ± 1.59	62.54 ± 11.51	21.19 ± 2.35
3a	27.72	0.9786	20.38	0.9850	16.90	0.9748	17.62 ± 4.72	18.67 ± 1.59	10.60 ± 2.07
3b	19.80	0.9784	24.75	0.9917	12.38	0.9805	29.70 ± 3.18	12.62 ± 2.54	13.29 ± 1.08
3c	20.38	0.9773	30.13	0.9987	21.66	0.9789	6.33 ± 0.51	10.68 ± 0.98	18.50 ± 3.87
3d	22.35	0.9775	21.00	0.9837	21.00	0.9805	9.16 ± 0.46	16.86 ± 3.76	13.29 ± 1.63
3e	25.67	0.9922	25.67	0.9829	26.65	0.9723	8.02 ± 0.88	37.16 ± 7.55	34.02 ± 5.90
3f	28.88	0.9827	24.75	0.9902	9.11	0.9904	11.35 ± 1.70	21.35 ± 3.47	8.91 ± 1.65
3g	21.00	0.9869	26.65	0.9912	12.16	0.9824	4.07 ± 0.38	14.88 ± 3.10	13.19 ± 5.15
AZA	46.75	0.9932	38.25	0.9890	-	-	30.74 ± 3.52	22.27 ± 1.56	-
TAC	-	-	-	-	25.78	0.9878	-	-	18.45 ± 2.12

The hCA I and hCA II isoenzymes were effectively inhibited by compounds 3a–g with IC50 values in the range of 19.80–28.88 nM (hCA I) and 21.00–30.13 nM (hCA II) while reference drug AZA had IC50 values as 46.75 nM (hCA I) and 38.25 (hCA II). Compounds 3a–g were found 1.6–2.4× and 1.3–1.9× more potent than reference AZA against hCA I and hCA II, respectively, by considering IC50 values.

Sulfamethazine-bearing compound 3g was the most effective inhibitor on hCA I isoenzyme, while sulfanilamide-bearing compound 3c was the most effective toward hCA II isoenzyme in terms of Ki values. The compounds 3a (1.7), 3c (4.8), 3d (3.3), 3e (3.8), 3f (2.7), and 3g (7.5) toward hCA I and 3a (1.2), 3b (1.8), 3c (2.1), 3d (1.3), and 3g (1.5) toward hCA II were more potent than AZA based on Ki values.

The most promising compound 3g having 4,6-dimethylpyrimidin-2-yl moiety was 7.5× and 12.4× more potent than AZA and compound 2, respectively. On the other hand, when the compound 3g was compared with 3e, substitution of the two methyl groups in 3g led to increasing activity that was 2× more potent. In addition, primary sulfonamide compound 3c was the second lead compound in regards to inhibitory potency on hCA I. Secondary amine derivatives, except 3g, were found less active than that of 3c which has sulfanilamide moiety.

Regarding hCA II, the most potent compound 3c having sulfanilamide moiety was 2× and 5.8× more potent than reference AZA and the intermediate compound 2, respectively. All of the secondary sulfonamide derivatives were less effective on hCA II than primary sulfonamide. Additionally, the compounds 3a–g can be considered as selective hCA I inhibitors according to Ki values. Furthermore, the second lead compound was 3b having sulfaguanidine moiety while pyrimidine ring bearing compounds 3e, 3f, and 3g were less effective than 3b.

### 3.3. Acetylcholinesterase inhibitory effects

AChE inhibitory potencies of the compounds 3a–g were presented in Table. When AChE inhibition potency of the compounds 3a–g were considered, IC50 values of the compounds were found in the range of 9.11–26.65 nM while their Ki values were recognized ranging between 8.91 ± 1.65 – 34.02 ± 5.90 nM. The reference compound tacrine had a IC50 value of 25.78 nM whereas its Ki value was 18.45 ± 2.12 nM. The compounds 3a–g were found as 1.2–2.8 fold more potent inhibitor s than reference drug Tacrine based on IC50 values (except 3e). In addition, the IC50 value of the cyclic compound 2 which has oxazole-5(4H)-ones was 38.50 nM.

Ki values of 3a–g were in the range of 8.91 ± 1.65 – 34.02 ± 5.90 nM. The compounds 3a–g were 1.4–1.7-fold more potent than the reference drug tacrine, except 3c and 3e. Compound 3f which has sulfamerazine moiety can be considered as a lead compound in AChE inhibition study with the lowest Ki (8.91 ± 1.65 nM) and IC50 (9.11 nM) values.

Based on the Ki values of the compounds, the intermediate 2-phenyl-4-(substituted benzylidene)oxazole-5(4 *H* )-ones (2) compound did not show significant inhibitory potency on hCA I, hCA II, and AChE enzymes with the highest Ki and IC50 values. Conversion of compound 2 which has oxazole-5(4H)-ones main skeleton to open-chain sulfonamide-based compounds 3a–g led to an increase in the enzyme inhibitory potency in the compounds. It can be expressed here that acid-catalyzed ring opening reaction led to having more potent bioactive compounds. So, benzamide moieties may cause an increase in bioactivity with all enzymes.

According to AChE enzyme inhibitory results of the compounds, converting intermediate compound 2 to final compounds moderately enhanced bioactivity with AChE, except compound 3e having sulfadiazine moiety. The most potent AChE inhibitor was compound 3f having sulfamerazine moiety in terms of Ki value. Among the pyrimidine bearing compounds 3e, 3f, and 3g, the compound 3f having one methyl group on pyrimidine ring was 1.5× more potent than dimethyl substituted derivative 3g while it was 3.8× more potent than unsubstituted derivative 3e. The substitution of secondary sulfonamide groups was found as a useful modification to increase AChE inhibition, except 3e.

## 4. Conclusion

In conclusion, a series of the compounds 3a–g were reported as novel enzyme inhibitors of hCA I, hCA II, and AChE. Enzyme inhibitory assays showed that novel benzamides 3a–g had inhibitory potencies against hCA I, hCA II, and AChE enzymes at the nanomolar levels. The Ki values of the compounds were calculated in the range of 4.07 ± 0.38 – 29.70 ± 3.18 nM for hCA I and 10.68 ± 0.98 – 37.16 ± 7.55 nM for hCA II, while the Ki values for AChE were calculated in the range of 8.91 ± 1.65 – 34.02 ± 5.90 nM. The most potent compounds 3g, 3c, and 3f can be considered as the leads of the study with promising results. The use of secondary sulfonamides can be considered as a useful modification to obtain more potent compounds towards AChE, while primary sulfonamide derivative generally had a better effect on hCA I and hCA II isoenzymes.
